# The Association Between Obesity Class and Cause‐Specific Mortality by Age and Sex in US Adults

**DOI:** 10.1111/cob.70104

**Published:** 2026-08-12

**Authors:** Amir Yazdanparast, Hala Tamim, Alison K. Macpherson, Jennifer L. Kuk

**Affiliations:** ^1^ School of Kinesiology and Health Science York University Toronto Canada

**Keywords:** body mass index, cause of death, mortality, obesity

## Abstract

To investigate differences in cause‐specific mortality risks between obesity classes stratified by age and sex, using a nationally representative sample of the US population. Differences in mortality risk by standard body mass index (BMI) categories with examination of Obesity Classes I–III, stratified by age (less or more than 65 years) and sex were examined using Cox proportional hazard ratios with data from NHANES Continuous (1999–2018) with mortality follow‐up through 31 December 2019. Among younger adults (< 65 years), Obesity Class III was associated with higher all‐cause, cardiovascular, non‐cardiovascular/non‐cancer and diabetes‐related mortality compared to Class I. Among older adults (≥ 65 years), there were fewer significant associations with only cardiovascular and diabetes‐related mortality risks being elevated in men with Obesity Class III and none in women. There were no differences in mortality risks between Obesity Classes I and II, except for diabetes‐related mortality in younger adults and older men. Obesity Class III is strongly associated with higher mortality risk in younger adults (< 65 years) and to a lesser degree in older adults (≥ 65 years). Mortality risks were generally similar between obesity Classes I and II. Therefore, younger adults under 65 years of age with severe obesity may benefit more from aggressive obesity management.

## Study's Importance

- What is already known about this subject?
  ○ Obesity increases the risk for a number of chronic diseases and all‐cause mortality risk. However, prior studies rarely examine the differences in cause‐specific mortality between obesity classes, particularly stratified by age and sex.
- What are the new findings in your manuscript?
  ○ Obesity class was positively associated with higher mortality risks in only younger adults, but the difference in mortality risk between Obesity Classes I and II did not reach statistical significance, with the exception of diabetes‐related mortality.
  ○ Obesity Class III is associated with higher all‐cause, cardiovascular, non‐cardiovascular/non‐cancer and diabetes‐related mortality compared to Class I, but only in younger adults (18–64 years), but not older adults (≥ 65 years).
- How might your results change the direction of research or the focus of clinical practice?
  ○ Younger adults (18–64 years) with Obesity Class III may benefit more from aggressive obesity management approaches, while the benefit for older adults (≥ 65 years) is less clear.
  ○ Although BMI is generally positively associated with several health risk factors and mortality outcomes, current BMI cut‐offs may not meaningfully differentiate the mortality risks between Obesity Classes I and II.

## 1 Introduction

Obesity is a significant public health concern as it negatively affects physical and psychological health, contributing to increased morbidity and mortality [1]. It has been well established that obesity is associated with several health complications, such as hypertension, glucose intolerance, Type II diabetes, dyslipidaemia, cardiovascular disease, coronary heart disease, stroke, dementia and some cancers [2, 3, 4]. Currently, obesity stands as the fifth leading global cause of death and is a contributor to the development of several significant causes of death such as cardiovascular diseases [5, 6], some cancers [7, 8] and other mortality outcomes [7, 9]. This has raised concerns that obesity and its complications might slow or even reverse the progress made in life expectancy over recent decades [4]. Notably, it is estimated that Obesity Class I may decrease life expectancy by 2–4 years, while Obesity Class III may reduce it by 8–10 years [10]. This suggests a trend of increasing mortality rates with increasing severity of obesity [11].

There is a substantial body of evidence indicating that obesity is associated with higher all‐cause mortality rates [2, 3, 7, 10, 11, 12, 13, 14, 15, 16]. However, the pattern of association between BMI and mortality rates may differ depending on the specific cause [3, 7, 10]. Most studies have examined obesity as a whole as compared to normal weight and did not examine differences between obesity sub‐classes as defined by the Center's for Disease Control and Prevention (CDC) [17]. As Obesity Class I is associated with elevated mortality risk as compared to normal weight, it is important to examine health and mortality differences specifically between the obesity sub‐classes. For example, it is reported that individuals with Class III obesity tend to have more physical limitations and difficulties with daily activities than those with Classes I and II obesity [18, 19]. Further, most studies did not examine differences in mortality risk for the obesity sub‐groups stratified by both age and sex, which is important given the clear differences in mortality risk for obesity between younger and older populations [9] and some studies reporting sex differences in how obesity relates with mortality risk [11]. Given that the suggested obesity therapies are often dependent upon the severity of obesity, it is important to examine the question of whether cause‐specific mortality rates may differ between obesity classes stratified by age and sex groups.

## 2 Methods

Data were obtained from adults over 20 years of age in the United States, participated in the National Health and Nutrition Examination Survey (NHANES) Continuous between 1999 and 2018 surveys with mortality follow‐up to 31 December 2019 (*n* = 55 081). A detailed explanation of the data collection methods has been previously reported [20, 21]. Prior to participating, all individuals provided written informed consent and the methods used were approved by the National Centre for Health Statistics [21]. The National Centre for Health Statistics used probabilistic record matching with death certificate data from the National Death Index (NCHS Linked Mortality File) until 31 December 2019, to obtain mortality information for participants in the NHANES Continuous survey. Primary causes of deaths were recorded as: diseases of the heart; malignant neoplasms; cerebrovascular diseases; chronic lower respiratory diseases; diabetes mellitus; accidents; Alzheimer's; influenza and pneumonia; nephritis, nephrotic syndrome and nephrosis; and other causes. Hypertension‐related and diabetes‐related deaths were classified if hypertension or diabetes were listed as one of the up to 20 contributing causes of death on the death certificate. Because the current analysis included only publicly available data, ethics approval from our institutional Research Ethics Board was not required.

Participants with missing education (*n* = 10 454), height, weight or BMI (*n* = 2634) or mortality follow‐up (*n* = 90) were excluded from the analysis, leaving a total of 41 903 patients included in the analysis. A trained technician conducted measurements of height and weight during the physical examination [21]. Standing height was measured using a fixed stadiometer and body weight was measured using an electronic load‐cell scale while wearing underwear, a paper gown and paper pants. Body mass index (BMI) was calculated [21] and categorised using CDC cut‐offs [22]: underweight—below 18.4 kg/m^2^; normal weight—18.5–24.9 kg/m^2^; overweight—25–29.9 kg/m^2^; Obesity Class I—30–34.9 kg/m^2^; Obesity Class II—35–39.9 kg/m^2^; Obesity Class III—40 kg/m^2^ and above.

Questionnaires were used to assess age, sex, smoking status, education level, ethnicity, physical activity and medication use [21]. Age was reclassified into two groups, younger adults (18–64 years of age) and older adults (65+ years of age). Educational level was recorded as: Less than 9th grade; 9‐11th grade (Including 12th grade with no diploma); High school graduate/GED or equivalent; Some college or Associate of Arts degree and College graduate or above and education was re‐categorised as the presence or absence of post‐secondary education (i.e., more than high school). The ethnic categories were collected as: Mexican American; Other Hispanic; non‐Hispanic White; non‐Hispanic Black and other non‐Hispanic race including non‐Hispanic multiracial and were re‐categorised as white and non‐white. Physical activity was self‐reported as the amount of moderate/vigorous leisure time physical activity over the past month. Physical activity status was dichotomised into those who engaged in any versus none. Participants were asked to report the prescription medications they took in the past month. When available, the interviewer asked to see the medication containers of all the products used. Medication names were matched to a Lexicon Plus database which contains medication ingredients, medication classifications and medication uses.

## 3 Statistical Analysis

The data from Continuous NHANES was weighted to be nationally representative of the United States population. The weighted means or prevalence with standard errors were calculated and reported within each age‐ and sex‐stratified BMI category. Due to the small sample size of the underweight BMI group, this group was excluded from the analysis of BMI group differences in mortality rates with Cox proportional hazards regression. Due to a violation in proportionality assumptions, the follow‐up times were truncated to 200 months. Cox proportional hazards ratio (HR) with consideration of competing interests using the Fine and Grey method was performed to assess differences in specific‐cause mortality risk by BMI classes with Class I obesity as the referent group. Mortality trends across obesity sub‐groups were also examined. Due to sample size, mortality causes were grouped into three larger categories. Mortality from diseases of the heart and cerebrovascular diseases were combined to represent mortality from cardiovascular diseases and all other deaths (chronic lower respiratory diseases; diabetes mellitus; accidents; Alzheimer's; influenza and pneumonia; nephritis, nephrotic syndrome and nephrosis and other causes), with the exception of malignant neoplasms, were combined to represent mortality from non‐cardiovascular/non‐cancer reasons. Diabetes and hypertension related mortality were also examined using Cox proportional hazards regression. HR models were adjusted for ethnicity, education, smoking and physical activity. In our supplemental analyses, additional adjustment for medications that were not used to directly treat the obesity outcome comorbidity (Type 2 diabetes, dyslipidaemia and hypertension medications) was done. For example, the hypertension related mortality models were not adjusted for hypertension medications but were adjusted for Type 2 diabetes and dyslipidaemia medications. The CVD models were only adjusted for Type 2 medications and the all‐cause, cancer and non‐CVD models were adjusted for all three medications. All results were stratified based on sex and age group.

All statistical analyses were performed using SAS software version 9.4 and a significance threshold of *p* < 0.05.

## 4 Results

Table 1 presents the participant characteristics. The sex and BMI class all‐cause mortality rates for young adults ranged between 2.4% and 13.2% and between 22.2% and 61.6% in older adults. All‐cause mortality rates in younger adults generally did not differ for most BMI categories as compared to Class I obesity. In younger adults (18–64 years), only Obesity Class III had a significantly higher prevalence of all‐cause mortality as compared to Class I obesity. Among older adults (≥ 65 years), the underweight and normal weight categories had the highest prevalence of all‐cause mortality, while Obesity Class I had lowest prevalence of mortality among older men and Obesity Class II had the lowest prevalence in older women.

**TABLE 1:** Characteristics of participants stratified by sex, age and BMI class.

	Underweight	Normal weight	Overweight	Obesity Class I	Obesity Class II	Obesity Class III
*Males under 65 years of age*						
*N* (*n*, %)	197.0 (1.3)	4115.0 (27.1)	5651.0 (37.2)	3202.0 (21.1)	1204.0 (7.9)	824.0 (5.4)
Age, years	35.1 ± 1.4*	37.7 ± 0.3*	42.3 ± 0.2	43.0 ± 0.3	42.7 ± 0.5	42.3 ± 0.5
BMI, kg/m^2^	17.6 ± 0.1*	22.6 ± 0.04*	27.4 ± 0.03*	32.2 ± 0.03	37.0 ± 0.1*	45.4 ± 0.2*
White ethnicity (%)	62.1 ± 4.5	65.0 ± 1.3	64.9 ± 1.4	65.5 ± 1.6	64.4 ± 2.0	63.9 ± 2.6
Higher education (%)	46.8 ± 4.5*	57.7 ± 1.5	59.5 ± 1.3	58.2 ± 1.0	58.8 ± 2.0	52.9 ± 2.2*
Smoker (%)	41.6 ± 4.6*	35.6 ± 1.3*	23.5 ± 0.8	22.9 ± 0.8	16.1 ± 1.2*	19.6 ± 1.7
Mortality rate (%)	13.2 ± 3.3*	4.5 ± 0.4	4.2 ± 0.3	4.8 ± 0.4	5.4 ± 0.8	8.7 ± 1.3*
Average follow‐up time, months	98.9 ± 6.6	107.4 ± 1.6*	106.0 ± 1.3*	98.6 ± 1.9	94.3 ± 2.6	88.6 ± 3.0*
*Females under 65 years of age*						
*N* (*n*, %)	340.0 (2.0)	4834.0 (29.0)	4553.0 (27.3)	3351.0 (20.1)	1908.0 (11.5)	1673.0 (10.0)
Age, years	36.1 ± 0.9*	39.5 ± 0.3*	42.3 ± 0.3*	43.5 ± 0.3	42.6 ± 0.4	42.9 ± 0.4
BMI, kg/m^2^	17.5 ± 0.1*	22.2 ± 0.03*	27.4 ± 0.03*	32.3 ± 0.03	37.2 ± 0.05*	46.0 ± 0.1*
White ethnicity (%)	72.9 ± 2.2*	69.1 ± 1.2*	62.8 ± 1.7*	58.5 ± 1.8	60.4 ± 1.8	58.1 ± 2.1
Higher education (%)	61.8 ± 3.6	70.9 ± 1.1*	63.5 ± 1.1	58.3 ± 1.4	55.3 ± 1.4	60.7 ± 1.4
Smoker (%)	35.0 ± 3.8*	21.1 ± 1.0	20.2 ± 0.8	20.6 ± 0.9	20.0 ± 1.1	18.8 ± 1.2
Mortality rate (%)	5.0 ± 1.5	2.6 ± 0.2	2.4 ± 0.3	3.0 ± 0.3	4.1 ± 0.6	5.2 ± 0.6*
Average follow‐up time, months	109.2 ± 4.2	108.6 ± 1.5*	104.4 ± 1.3	101.2 ± 1.5	102.3 ± 2.0	92.8 ± 2.6*
*Males over 65 years of age*						
*N* (*n*, %)	63.0 (1.3)	1292.0 (25.9)	2064.0 (41.4)	1082.0 (21.7)	351.0 (7.0)	134.0 (2.7)
Age, years	73.1 ± 0.9	73.7 ± 0.3*	73.0 ± 0.2*	72.2 ± 0.2	70.9 ± 0.4*	70.4 ± 0.6*
BMI, kg/m^2^	17.3 ± 0.1*	22.9 ± 0.1*	27.4 ± 0.04*	32.1 ± 0.1	37.0 ± 0.1*	43.8 ± 0.6*
White ethnicity (%)	63.9 ± 7.7*	72.6 ± 1.8*	82.0 ± 1.2	81.9 ± 1.4	86.5 ± 1.9	77.3 ± 4.3
Higher education (%)	48.2 ± 9.5	55.6 ± 2.2	56.0 ± 1.5	54.1 ± 2.4	56.2 ± 4.0	54.3 ± 5.1
Smoker (%)	55.5 ± 9.6*	15.8 ± 1.4*	10.1 ± 1.0*	6.1 ± 0.9	10.2 ± 2.3	7.7 ± 4.3
Mortality rate (%)	49.2 ± 9.1*	39.2 ± 1.8*	32.1 ± 1.5	30.8 ± 1.8	31.8 ± 3.7	35.7 ± 5.8
Average follow‐up time, months	64.1 ± 6.2*	78.6 ± 2.0	80.4 ± 2.0	77.2 ± 2.4	76.7 ± 3.8	67.4 ± 5.5
*Females over 65 years of age*						
*N* (*n*, %)	85.0 (1.7)	1361.0 (26.9)	1657.0 (32.7)	1126.0 (22.2)	536.0 (10.6)	300.0 (5.9)
Age, years	74.7 ± 0.8*	74.4 ± 0.2*	73.8 ± 0.2*	73.1 ± 0.2	71.9 ± 0.3*	71.5 ± 0.4*
BMI, kg/m^2^	17.2 ± 0.2*	22.5 ± 0.1*	27.4 ± 0.1*	32.1 ± 0.1	37.0 ± 0.1*	44.6 ± 0.4*
White ethnicity (%)	85.2 ± 2.8*	82.3 ± 1.1*	79.8 ± 1.4	77.7 ± 1.7	74.0 ± 2.2	75.4 ± 2.6
Higher education (%)	46.3 ± 6.5	52.1 ± 1.9*	47.0 ± 1.8	46.7 ± 1.8	44.8 ± 2.9	47.7 ± 4.2
Smoker (%)	21.0 ± 5.4*	9.9 ± 1.0*	7.4 ± 0.8	5.5 ± 0.8	4.8 ± 1.2	94.7 ± 1.6
Mortality rate (%)	61.6 ± 6.7*	35.5 ± 1.6*	28.3 ± 1.5	27.6 ± 1.7	22.2 ± 2.0*	28.0 ± 3.0
Average follow‐up time, months	78.3 ± 6.8	83.7 ± 2.1	84.0 ± 1.7	82.5 ± 2.3	79.8 ± 2.9	81.9 ± 3.8

*Note:* Values reported are means ± standard error or prevalence (%) ± standard error.

Abbreviation: BMI = body mass index.

* Significantly different from Obesity Class I (*p* < 0.05).

**FIGURE 1:**
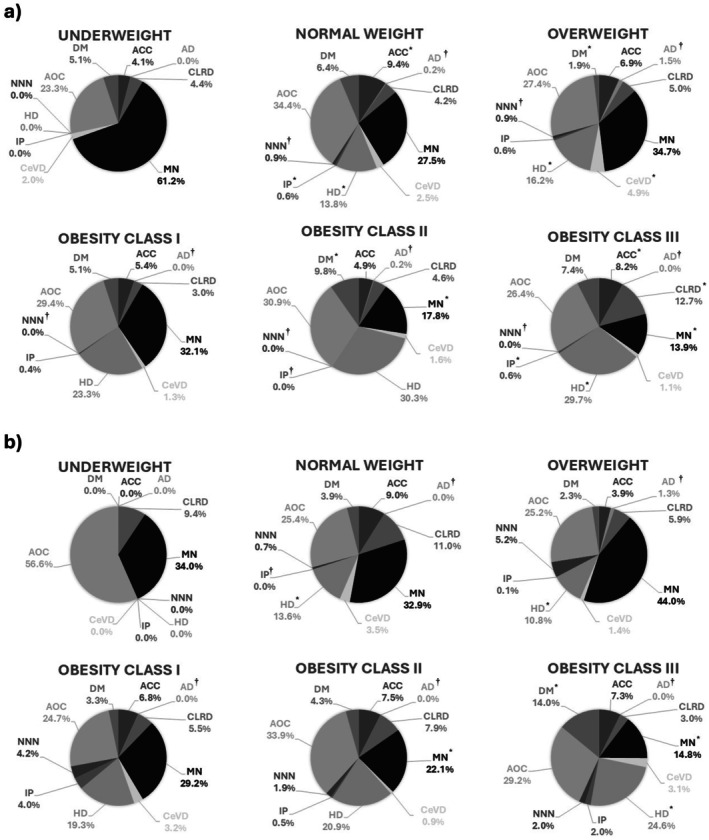
Differences in prevalence of mortality due to a specific cause among (a) younger men and (b) younger females (18–64 years), by BMI class. Values represent weighted prevalence. ACC = accidents; AD = Alzheimer's disease; AOC = all other causes; CeVD = cerebrovascular diseases; CLRD = chronic lower respiratory diseases; DM = diabetes mellitus; HD = diseases of heart; IP = influenza and pneumonia; MN = malignant neoplasms; NNN = nephritis, nephrotic syndrome and nephrosis. *Significant difference Obesity Class I. ^†^Unable to analyse the difference due to zero observations in that cause of death‐BMI category group.

Figure 1 shows the unadjusted prevalence of the various causes of mortality among younger men and women by BMI class. Malignant neoplasms and diseases of the heart were the most common causes of mortality among younger adults (18–64 years, Figure 1a,b). The specific significant BMI class differences in the prevalence of the various cause‐specific mortality types varied by mortality type, but with most following a similar pattern of association. In general, normal weight and overweight groups were at a lower mortality risk than Obesity Class I, while Obesity Class III had higher mortality rates, with the exception of malignant neoplasms mortality that was not associated with BMI among younger adults (18–64 years, *p* < 0.05, Figure 1a). In younger men, Obesity Class II had a greater prevalence of deaths due diabetes mellitus (9.8% vs. 5.15%; *p* < 0.001) but a lower prevalence of death due to malignant neoplasms (17.8% vs. 32.1%; *p* < 0.001) compared to Class I, while Class III was associated with a higher prevalence of death due to diseases of the heart (29.7% vs. 23.3%; *p* < 0.02), chronic lower respiratory diseases (12.7% vs. 3.0%; *p* < 0.001) and lower prevalence of death due to malignant neoplasms (13.9% vs. 32.1%; *p* < 0.001, Figure 1a) than Class I. In younger women (18–64 years), Obesity Class III was associated with a higher prevalence of death due to diabetes mellitus (14.0% vs. 3.3%; *p* < 0.001) and diseases of the heart (24.6% vs. 19.3%; *p* < 0.01), while Obesity Class II was only associated with a lower prevalence of death due to malignant neoplasms compared to Class I (14.8% vs. 29.2%; *p* < 0.001, Figure 1b).

**FIGURE 2:**
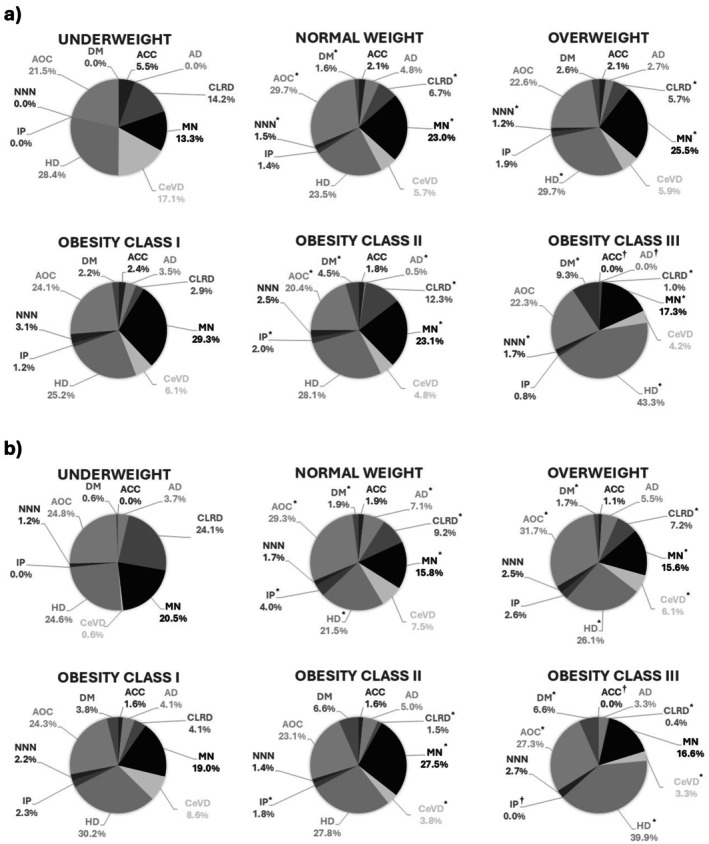
Differences in prevalence of mortality due to a specific cause among (a) older men and (b) older females, (≥ 65 years) by BMI class. Values represent weighted prevalence. ACC = accidents; AD = Alzheimer's disease; AOC = all other causes; CeVD = cerebrovascular diseases; CLRD = chronic lower respiratory diseases; DM = diabetes mellitus; HD = diseases of heart; IP = influenza and pneumonia; MN = malignant neoplasms; NNN = nephritis, nephrotic syndrome and nephrosis. *Significant difference Obesity Class I. ^†^Unable to analyse the difference due to zero observations in that cause of death‐BMI category group.

Figure 2 shows the unadjusted prevalence of the various causes of mortality among older men and women (≥ 65 years) by BMI class. The most common causes of death among older adults were also malignant neoplasms and diseases of the heart (Figure 2a,b). Older men with Obesity Class II had a significantly higher prevalence of death due to diabetes mellitus (4.5% vs. 2.2%; *p* < 0.001) and chronic lower respiratory diseases (12.3% vs. 2.9%; *p* < 0.001) and had a lower prevalence of death due to malignant neoplasms (23.1% vs. 29.3%; *p* < 0.01), compared to Obesity Class I (Figure 2a). Older women (≥ 65 years) with Obesity Class II had a higher prevalence of death due to malignant neoplasms (27.5% vs. 19.0%; *p* < 0.001) and lower prevalence of death due to chronic lower respiratory diseases (1.5% vs. 4.1%; *p* < 0.05) compared to Class I. Both older men and women with Obesity Class III had a significantly higher prevalence of death due to diseases of the heart (men: 43.3% vs. 25.2%; *p* < 0.001; women: 39.9% vs. 30.2%; *p* < 0.001) and diabetes mellitus (men: 9.3% vs. 2.2%; *p* < 0.05; women: 6.6% vs. 3.8%; *p* < 0.05, Figure 2a,b). Older men (≥ 65 years) had a lower prevalence of death due to malignant neoplasms (17.3% vs. 29.3%; *p* < 0.001) and chronic lower respiratory diseases (1.0% vs. 2.9%; *p* < 0.002), compared to those with Class I.

**FIGURE 3:**
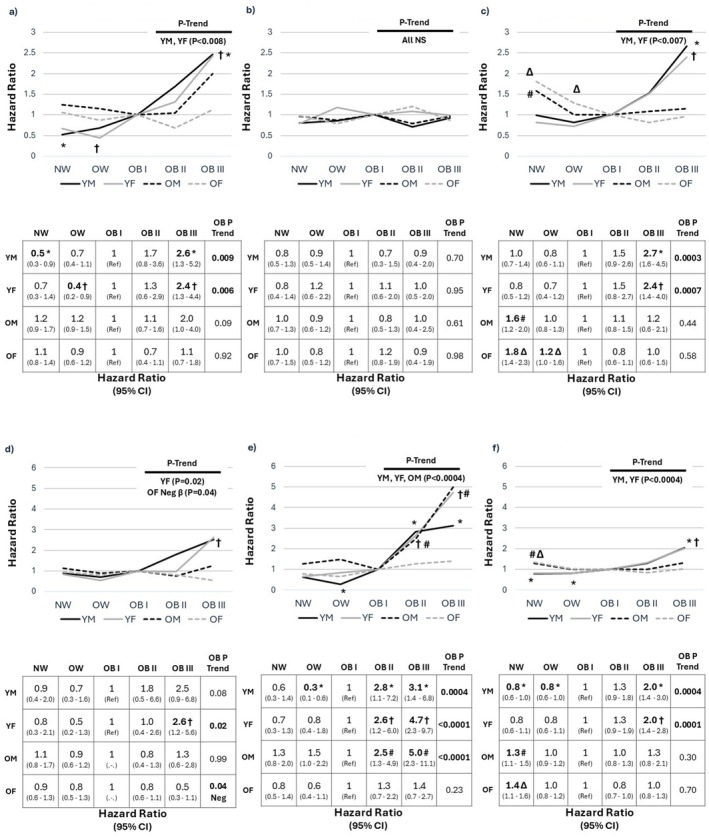
Differences in hazard ratios of mortality due (a) cardiovascular diseases, (b) malignant neoplasms, (c) non‐cardiovascular diseases/non‐cancer causes, (d) hypertension‐related causes, (e) diabetes‐related causes and (f) all‐cause by age, sex and BMI class. Values represent adjusted hazard ratios adjusted for ethnicity, education, smoking and physical activity. NW = normal weight; OB I = Obesity Class I; OB II = Obesity Class II; OB III = Obesity Class III; OF = older females; OM = older males; OW = overweight; YF = younger females; YM = younger males. *Significant difference from younger males (18–64 years) with obesity class I. ^†^Significant difference from younger females with Obesity Class I. ^#^Significant difference from older males (≥ 65 years) with Obesity Class I. ^∆^Significant difference from older females with Obesity Class I.

Figure 3 shows the differences in hazard ratios of mortality due to: cardiovascular diseases (Figure 3a), malignant neoplasms (Figure 3b), non‐cardiovascular/non‐cancer causes (Figure 3c) hypertension‐related causes (Figure 3d), diabetes‐related causes (Figure 3e) and all‐cause (Figure 3f) mortality by age group, sex and BMI class adjusted for ethnicity, education, smoking and physical activity. The HR for all‐cause mortality was positively associated with obesity sub‐class in younger individuals (18–64 years, *p* < 0.0004). Both younger men and women with Obesity Class III had a higher risk for all‐cause mortality compared to those with Obesity Class I (HR = 2.1, *p* < 0.05, Figure 3f). There were no significant group differences or trends in all‐cause mortality by obesity sub‐class in older men or women (≥ 65 years, *p* > 0.10). There were no significant differences in mortality risk between obesity classes among older men or women (*p* > 0.05). This suggest that severe obesity may be a significant risk factor among younger individuals (18–64 years), but not older adults (≥ 65 years).

A positive association was also observed between obesity sub‐class and cardiovascular mortality among younger adults (18–64 years, *p* < 0.009, Figure 3a). Younger men and women with Obesity Class III had significantly higher cardiovascular mortality risk, respectively, compared to those with Obesity Class I (HR = 2.4–2.6, *p* < 0.0001, Figure 3a). No trends or group differences were observed between obesity classes among older men or women (*p* > 0.05). This suggests that within obesity classes, younger, but not older adults with severe obesity are at significantly higher risk of cardiovascular mortality.

In the current study, no significant associations or group differences between obesity sub‐class and mortality due to malignant neoplasms were observed in either younger or older adults (*p* > 0.30, Figure 3b). Among younger adults a positive association between obesity sub‐class and non‐cardiovascular/non‐cancer mortality was observed (18–64 years, *p* < 0.007). There were also differences between obesity classes with mortality risk due to non‐cardiovascular/non‐cancer causes being approximately 2.7 and 2.4 times higher for younger men and women with Obesity Class III, respectively, compared to Obesity Class I (*p* < 0.05, Figure 3c). Conversely, there were no significant associations or group differences between obesity sub‐class and non‐cardiovascular/non‐cancer mortality observed among older adults (≥ 65 years, *p* > 0.24). However, mortality risk due to non‐cardiovascular/non‐cancer causes was not different between obesity sub‐classes, but normal weight was at higher risk for non‐cardiovascular/non‐cancer mortality (*p* > 0.24, Figure 3c). This suggests that severe obesity is a significant risk factor for non‐cardiovascular/non‐cancer mortality among younger individuals (18–64 years), but not older adults (≥ 65 years).

Mortality risk for death due to hypertension‐related causes was positively related with obesity sub‐groups among younger (18–64 years) women (*p* = 0.02), but not men (*p* = 0.08). Only younger women with Obesity Class III had a significantly higher hypertension‐related mortality risk compared to those with Class I obesity (*p* = 0.01, Figure 3d). However, no differences were observed between obesity classes in younger men (*p* > 0.07, Figure 3d). Among older men (≥ 65 years), there were no significant associations or group differences in hypertension‐related mortality for obesity sub‐groups (*p* > 0.33). Among older women, there was a negative association between obesity sub‐class and hypertension‐related mortality (*p* = 0.04), but no significant group differences between obesity classes were observed (*p* > 0.10). This suggests that severe obesity might be a more significant risk factor for hypertension‐related mortality among younger women than men and could be protective in older women.

A positive association between obesity sub‐class and diabetes‐related mortality risk was observed in younger adults and older men (*p* < 0.0004). Younger men and women with Obesity Classes II and III had a higher mortality risk due to diabetes‐related causes compared to those with Class I obesity (18–64 years, HR = 2.6–4.7, *p* < 0.03, Figure 3e). Similarly, older men with Obesity Classes II and III had a higher diabetes‐related mortality risk compared to those with Class I obesity (≥ 65 years, HR = 2.5–5.0, *p* < 0.009, Figure 3e). No significant differences or trends were observed for obesity classes and diabetes‐related mortality in older women (*p* > 0.32, Figure 3e). This suggests that obesity is associated with step‐increases in diabetes mortality risk with increasing BMI category among younger adults (18–64 years) as well as older men (≥ 65 years), but not older women.

When the analyses were further adjusted for certain obesity‐related medication use fewer of the associations between Obesity Class II and III were significantly associated with the various causes of mortality (Figure S1). Within younger men (18–64 years), Obesity Class III was associated with three instead of four of the six mortality outcomes with additional adjustment for medication use. In younger women, only two of the previously five significant associations between Obesity Class III and the six mortality outcomes remained significant with additional adjustment for medication use. In older adults, the associations between Obesity Class III and the mortality outcomes were unchanged with additional adjustment for medication use. For Class II obesity, there were zero or one significant association prior to and after adjustment for medication use for younger adults and older men. In older women, there were no significant associations prior to adjusting for medication use and two negative associations with Class II obesity and mortality risk after adjustment for medication use.

## 5 Discussion

This study examined the differences in mortality risks between BMI classes, stratified by age and sex. There were very few differences in mortality risks between Classes I and II obesity, while Class III is associated with significantly higher mortality compared to Class I in younger adults (18–64 years) and with very few differences in mortality risk by obesity class in older adults (≥ 65 years). Though there were subtle variations between the various causes of death, Obesity Class III was associated with higher mortality risk for cardiovascular, non‐cardiovascular/non‐cancer, diabetes‐related and all‐cause mortality causes in younger adults. Severe obesity was less consistently associated with mortality risk in older adults.

Obesity has been shown to be associated with significantly higher all‐cause mortality within different populations, with some suggesting a J‐shaped or linear pattern of association [2, 3, 7, 10, 11, 12, 13, 14, 15, 16]. However, to our knowledge, no other study directly examined differences between the obesity classes, while simultaneously stratifying by both age and sex. Other studies have generally used normal weight as reference group and did not examine the differences between obesity classes [11, 13]. As Obesity Class I is generally associated with increased mortality risk as compared to normal weight [11], it is important to directly examine whether there are differences in risk between obesity sub‐classes in order to substantiate the appropriateness of current BMI obesity class cut‐points. In the current study, it was observed that all‐cause mortality was positively related to BMI only in younger adults. While there were no differences in all‐cause mortality between Classes I and II obesity, Obesity Class III was associated with significantly higher all‐cause mortality risk compared to Obesity Class I. Some studies have previously examined the association between all‐cause mortality and BMI using models stratified by either sex or age, but not both simultaneously [7, 11, 13]. This may be important as we observe clear differences in how BMI is associated with all‐cause mortality between younger and older adults and to a lesser degree sex. Some studies report elevated all‐cause mortality risk in men with Obesity Class II and women with Obesity Classes II and III compared to those with normal weight [13], while others report elevated all‐cause mortality risks for both men and women for all obesity classes compared to normal weight [11]. However, these studies did not directly examine the differences between obesity classes. Among older adults, the results have been more divided with some studies reporting no association [2, 13, 14, 23], a negative association [24] and positive association between all‐cause mortality risk and BMI [11, 16]. We also observe no association between all‐cause mortality and BMI in older men or women (≥ 65 years). This suggests that severe obesity is a significant risk factor for all‐cause mortality among younger individuals, but this association may change with age.

Cardiovascular deaths are one of the leading causes of death in the world [6]. Some studies report elevated cardiovascular mortality with overweight and obesity [3, 10, 11, 15, 25], while others report cardiovascular mortality is only elevated in Obesity Classes II and III compared to normal weight [13]. One study suggests the significantly higher cardiovascular mortality risk with Obesity Classes II and III as compared to normal weight is consistent between middle age (aged 45–64 years old) and older adults (older than 65 years) [13]. However, no study has directly examined differences in cardiovascular mortality risk between the obesity classes, stratified by sex and age. In this study, no differences in cardiovascular risk between Classes I and II were observed in younger or older adults, while only Obesity Class III was associated with significantly higher risk of cardiovascular mortality in younger adults, but not older adults.

Elevated cancer mortality rate has also been positively associated with BMI and obesity [3, 4, 7, 8, 15, 26, 27]. However, again the no other study directly examined differences in cancer mortality risk between the obesity classes, stratified by sex and age. Some studies report a J‐shape relationship between BMI and cancer mortality risk [7, 11], while others found no association between obesity and cancer mortality risk [9, 28, 29, 30]. We also observed no association between BMI and cancer mortality in both younger and older men and women. Even within studies that report a significant association between obesity and the individual cancer mortality types suggest a relatively lower mortality risk (hazard ratio < 2.0) compared to other causes of death [7, 11]. Further, while obesity is positively associated with some cancers such as breast [7, 8, 27], liver [7, 8], uterine [7, 27], prostate [7] and colorectal cancers [7, 8, 27], it is not associated with other cancers such as ovarian [27] and brain [7, 8, 27] and is negatively associated with other cancers such as melanoma [27] and lung cancer [7, 8, 27]. Therefore, further research, with larger sample sizes may be necessary to fully understand differences in cancer mortality risk and the specific cancer sub‐types between obesity classes, among younger and older men and women.

A number of studies have investigated the association between non‐cardiovascular/non‐cancer mortality risks with BMI [9, 23, 24, 30]. Some studies suggest higher mortality risk with obesity in younger adults compared to normal weight [9]. In the present study, younger adults with Class III obesity had significantly higher non‐cardiovascular/non‐cancer mortality risk compared to Class I, but with no differences with Class II. This category of mortality is more heterogeneous in nature with causes that include chronic lower respiratory diseases; diabetes mellitus; accidents; Alzheimer's; influenza and pneumonia; nephritis, nephrotic syndrome and nephrosis and other causes. These causes of death vary in the strength of their association with obesity, with some causes being strongly associated (i.e., diabetes, influenza and pneumonia and respiratory diseases, nephritis, nephrotic syndrome and nephrosis), while others such as Alzheimer's disease, neurological, accidents and self‐harm causes of death are less strongly or not related [7]. Among older adults (≥ 65 years), no differences in non‐cardiovascular/non‐cancer mortality risks were observed between obesity classes, while normal weight was associated with a significantly higher mortality risk. Other studies have also suggested higher non‐cardiovascular and/or non‐cancer mortality risk in older adults with normal weight, compared to those with overweight and obesity [23, 24]. The lower mortality risk observed in older adults with higher BMIs may be due to the protective effects of excess body fat [24] or higher fat‐free mass that is also associated with higher BMIs [31], also known as the obesity paradox. Indeed, although obesity is associated with higher risk for developing certain morbidities, it is also associated with higher survival rates for those same conditions [32]. The concept of a survival advantage or lack of negative mortality outcomes for older adults with obesity has been demonstrated with other measures of obesity such as waist circumference and body fat as assess using sum or skinfolds [33]. Further, the elevated non‐cardiovascular/non‐cancer mortality risk among those with normal weight may be reflective of the underlying frailty in this population [23]. It is important to consider that weight loss is not only associated with loss of fat mass, but also lean mass and skeletal muscle and could be particularly detrimental in older populations who are at greater risk for sarcopenia and frailty [34]. Thus, preservation of both fat and lean mass may provide a survival advantage that is unique to older age. This suggests that younger adults may benefit more from aggressive obesity management and weight loss strategies, while older adults may be better served with weight maintenance approaches.

Hypertension and Type II diabetes are comorbidities that are strongly associated with obesity and contribute to the development of cardiovascular disease mortality risk [6, 35]. In this study, we only observe Obesity Class III to be associated with hypertension‐related mortality risk in younger women. Type II diabetes is one of the most strongly obesity‐related comorbidities and this was the only mortality outcome where we observed a significantly higher mortality risk for Obesity Class II as compared to Obesity Class I in younger adults. The association between obesity and Type II diabetes mortality outcomes was not as clear in older adults. A number of studies have examined the association between BMI and all‐cause mortality in patients with diabetes; however the results have been inconsistent. One study reports that patients with Type II diabetes and Obesity Classes I, II and III did not have significantly different mortality risk as compared to normal weight patients with type II diabetes, though they may have been underpowered as the sample size of the deaths in the obesity sub‐classes were relatively small (*n* = 1–69) [36]. Other studies report higher diabetes mortality rates in Obesity Class III compared to Classes I [37, 38] or II [37] among those with Type II diabetes. Conversely, another study found a negative association between BMI and mortality risk among patients with Type II diabetes, despite having a predominating middle‐aged sample [39]. However, these studies also lacked age and sex stratification, which leaves the question of whether these associations differ between younger and older men and women. In the current analyses, we only observed a significant association between diabetes‐related mortality risk and Classes II and III obesity in older men, but not women. This suggests that there may be sex differences in how obesity relates with certain mortality risk outcomes particularly in older adults.

Our results are not consistent as to whether there are sex differences in how severe obesity may relate to mortality risk in men as compared to women. For most of the mortality outcomes, the hazard ratios for Obesity Class III were similar between men and women. For diabetes‐related mortality, the associations were bit stronger in younger women than men; however, the reverse was true in older men. For hypertension‐related mortality, obesity sub‐class was only significantly associated in women, with a positive association in younger women and a negative association in older women. Although women have higher rates of obesity and a greater impact of obesity on health‐related quality of life [40], obesity‐related cancers [41] and mental health disorders [41], they have a proportionally lower risk for obesity‐related cardiometabolic risk [41, 42]. This may be due to the lower levels of visceral fat in women as compared to men for a given BMI or waist circumference [43, 44]. Alternatively, these sex differences may be related to differences in hormonal factors, health behaviours or medical care utilisation [41]. It is suggested that women are less likely to have negative health‐related lifestyle risk factors [42] and are more likely to seek health care than men [45]. Further research is needed to clarify the sex differences in obesity health risk and whether sex‐specific management guidelines are needed.

Across the various causes of mortality, we observed a significant association between obesity class and mortality risk for most outcomes in younger adults (18–64 years); there were minimal statistically significant differences between Classes I and II obesity, while Class III was more consistently associated with significantly higher mortality risk in younger adults than older adults. Though BMI is generally positively associated with several health risk factors and many of the mortality outcomes examined here, it is unclear whether Obesity Classes I and II cut‐offs discriminate two distinctly different health risk groups that require differentiation. This could perhaps indicate that the mortality risk associated with obesity is not linear, but with more gradual increase in risk between Classes 1 and II with a more exponential increase in risk with Class III obesity as seen with cardiovascular, non‐cardiovascular/non‐cancer and hypertension related mortality outcomes in this study. Obesity is associated with several comorbidities and those with severe obesity are more likely to have multiple comorbidities [46] and this greater mortality risk could potentially reflect an exacerbated additive effect of having multiple comorbidities in Class III. In our supplementary analyses, we present our analyses with additional adjustment for medication use. As the development of obesity related comorbidities would fall on the causal pathway for how obesity relates with mortality risk, adjustment for medication use would likely be an overadjustment and explain why many of the significant associations between obesity and mortality outcomes were no longer significant after adjustment for medication use. To address this in part, we did not adjust for the medications that would directly be relevant to the mortality outcomes examined. For example, we did not adjust for diabetes medication use for the diabetes‐related mortality models and only adjusted for hypertension and dyslipidaemia medication use. Nevertheless, obesity comorbidities tend to cluster together and may explain why many of the associations for the mortality outcomes were diminished but remained associated in younger adults even after adjustment for medication use. Indeed, BMI cut‐offs have been revised over time to reflect the changing association between BMI and health risk over time. There is evidence to suggest that the risk for certain obesity comorbidities such as hypertension and dyslipidaemia may be decreasing over time, while the risk for diabetes may be increasing [47]. Additionally, advancement of medicine, better screenings and more effective treatments may also affect the prevalence of these chronic conditions associated with obesity and ultimately affect the associated mortality risks [47]. This study provides evidence to suggest that mortality outcomes are similar between Obesity Classes I and II, while Obesity Class III is clearly associated with elevated mortality risk and may require more aggressive obesity management approaches. More research is necessary to determine the most currently appropriate BMI cut‐offs to delimitate obesity sub‐classes that differ in health risk.

### 5.1 Strengths and Limitations

One notable strength of this study is a large, weighted sample to reflect the United States population. However, this study has several limitations that should be taken into account. BMI was measured only at the baseline. Recent and previous weight loss or gain, as well as body shape may also alter the association between BMI and mortality and was not considered in this study [16]. While weight gain is generally associated with increased mortality risk [2] the association with weight loss is less clear. Some research has suggested that intentionality of weight loss may be important when considering the association with mortality as intentional weight loss is reflective of purposeful weight loss behaviours while unintentional weight loss may be more reflective of underlying disease and worse health. Thus, only unintentional weight loss may be associated with greater mortality risk. Conversely, other studies report that both intentional and unintentional weight loss are associated with increased mortality risk. While short‐term studies examining differences in metabolic risk are clearly associated with improvements in metabolic risk factors, it is not clear why these may not translate into reductions in mortality risk. The prospective cohort design of the study with mortality follow‐up and lack of follow up BMI measurements, limits the ability infer casual association between the variables. Consequently, further research is necessary to explore the relationship between BMI classes and mortality, in different sub populations, such as patients with pre‐existing conditions. Despite our large sample size, we were unable to examine some of the less common causes of death that were lumped into the heterogeneous ‘non‐cardiovascular/non‐cancer’ category and is subject to higher Type II error. Future research will be needed to confirm our observations with this less common causes of mortality. Finally, as an exploratory analyses, the large number of comparisons increases the likelihood of Type I errors.

Among younger adults, Obesity Class III is generally associated with the highest mortality risks, while there were minimal differences between Obesity Classes I and II. This study contributes to the growing literature that suggests that obesity is associated with greater mortality risk in younger than older individuals. However, whether there are sex differences is unclear and may differ by age group and the comorbidity in question. Further research is needed to better understand the age and sex differences in obesity health risk. Thus, these differences should be considered when designing obesity management strategies, as they highlight the need for more aggressive approaches for these populations.

## Author Contributions

The authors had full access to all the data in the study and take responsibility for the integrity of the data and the accuracy of the data analysis. A.Y. wrote the manuscript and analysed the data. H.T., A.K.M. and J.L.K. revised the manuscript for important intellectual content. All authors contributed to the concept and design of the study, interpretation of the data and approved the final submitted version of the manuscript.

## Funding

The authors have nothing to report.

## Conflicts of Interest

The authors declare no conflicts of interest.

## Supporting information


**Figure S1:** Differences in hazard ratios of mortality due (a) cardiovascular diseases, (b) malignant neoplasms, (c) non‐cardiovascular diseases/non‐cancer causes, (d) hypertension‐related causes, (e) diabetes‐related causes and (f) all‐cause by age, sex and BMI class.

## Data Availability

The data that support the findings of this study are available in NHANES at https://wwwn.cdc.gov/nchs/nhanes/. These data were derived from the following resources available in the public domain: ‐ Continuous NHANES, https://wwwn.cdc.gov/nchs/nhanes/continuousnhanes/default.aspx.
