# Supplementary material for: The Association Between Obesity Class and Cause‐Specific Mortality by Age and Sex in US Adults
Source: Clin Obes. 2026 Aug 12;16(5):e70104. doi: 10.1111/cob.70104 (PMC13469556; doi:10.1111/cob.70104)
Supplement: Supplementary file 1 [file COB-16-e70104-s001.pdf]

The Association Between Obesity Class and Cause-Specific Mortality by Age and Sex in U.S. Adults.

Amir Yazdanparast MFSc,[1] Hala Tamim Ph.D,[1] Alison K. Macpherson Ph.D,[1] Jennifer L. Kuk, Ph.D.[1]

[1] School of Kinesiology and Health Science, York University, Toronto, Canada;

Address for correspondence:
Jennifer L Kuk
York University
Sherman Health Science Research Centre, Room 2002
4700 Keele Street
Toronto, ON M3J 1P3
Telephone: (416) 736-2100 x20080
Fax: 416-736-5772
E-mail: jennkuk@yorku.ca

Supplemental Figure. Differences in hazard ratios of mortality due to a) cardiovascular diseases, b) malignant neoplasms, c) non-cardiovascular diseases/non-cancer causes, and d) hypertension-related causes, e) diabetes-related causes, and f) all-cause by age, sex, and BMI class.

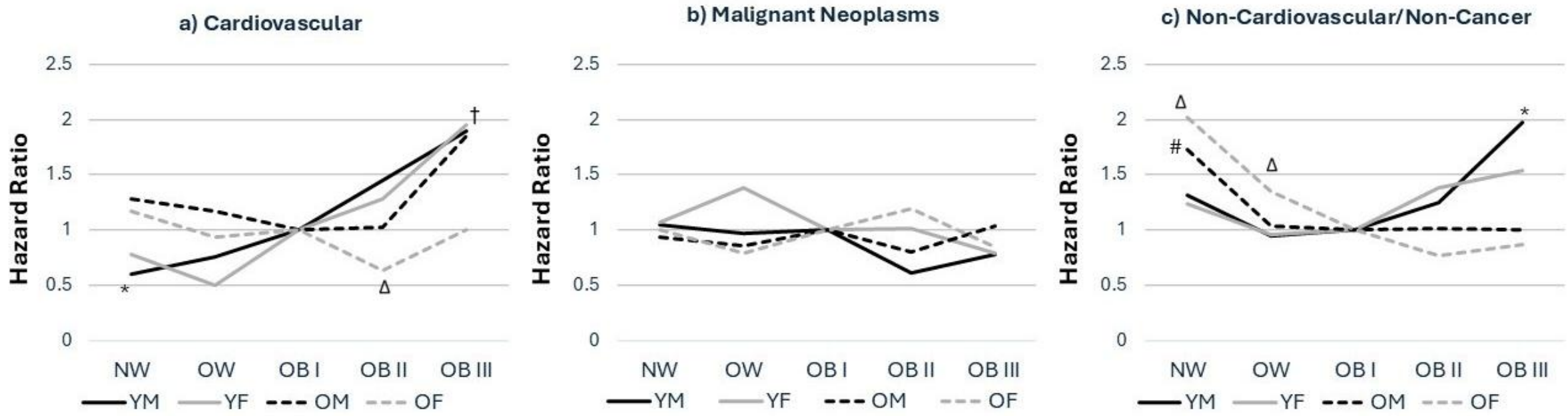

a) Cardiovascular

	NW	OW	OB I	OB II	OB III
YM	**0.6 *** (0.4 - 0.98)	0.8 (0.5 - 1.3)	1 (. - .)	1.4 (0.7 - 3.1)	1.9 (0.9 - 3.9)
YF	0.8 (0.4 - 1.6)	0.5 (0.2 - 1.0)	1 (. - .)	1.3 (0.6 - 2.8)	**1.9†** (1.1 - 3.6)
OM	1.3 (0.9 - 1.7)	1.2 (0.9 - 1.6)	1 (. - .)	1.0 (0.7 - 1.6)	1.8 (0.9 - 3.8)
OF	1.2 (0.9 - 1.6)	0.9 (0.7 - 1.3)	1 (. - .)	**0.6 Δ** (0.4 - 0.96)	1.0 (0.6 - 1.6)

Hazard Ratio (95% CI)

b) Malignant Neoplasms

	NW	OW	OB I	OB II	OB III
YM	1.1 (0.7 - 1.6)	1.0 (0.6 - 1.6)	1 (. - .)	0.6 (0.3 - 1.3)	0.8 (0.4 - 1.7)
YF	1.1 (0.6 - 2.0)	1.4 (0.7 - 2.6)	1 (. - .)	1.0 (0.5 - 1.9)	0.8 (0.4 - 1.6)
OM	0.9 (0.7 - 1.3)	0.9 (0.6 - 1.2)	1 (. - .)	0.8 (0.5 - 1.3)	1.0 (0.4 - 2.8)
OF	1.0 (0.7 - 1.5)	0.8 (0.5 - 1.2)	1 (. - .)	1.2 (0.8 - 1.9)	0.8 (0.4 - 1.9)

Hazard Ratio (95% CI)

c) Non-Cardiovascular/Non-Cancer

	NW	OW	OB I	OB II	OB III
YM	1.3 (0.9 - 1.9)	0.9 (0.7 - 1.3)	1 (. - .)	1.2 (0.7 - 2.1)	**2.0 *** (1.2 - 3.2)
YF	1.2 (0.8 - 1.9)	1.0 (0.6 - 1.5)	1 (. - .)	1.4 (0.8 - 2.5)	1.5 (0.9 - 2.6)
OM	**1.7#** (1.3 - 2.2)	1.0 (0.8 - 1.3)	1 (. - .)	1.0 (0.7 - 1.4)	1.0 (0.6 - 1.8)
OF	**2.0 Δ** (1.6 - 2.6)	**1.3 Δ** (1.1 - 1.7)	1 (. - .)	0.8 (0.6 - 1.1)	0.9 (0.6 - 1.3)

Hazard Ratio (95% CI)

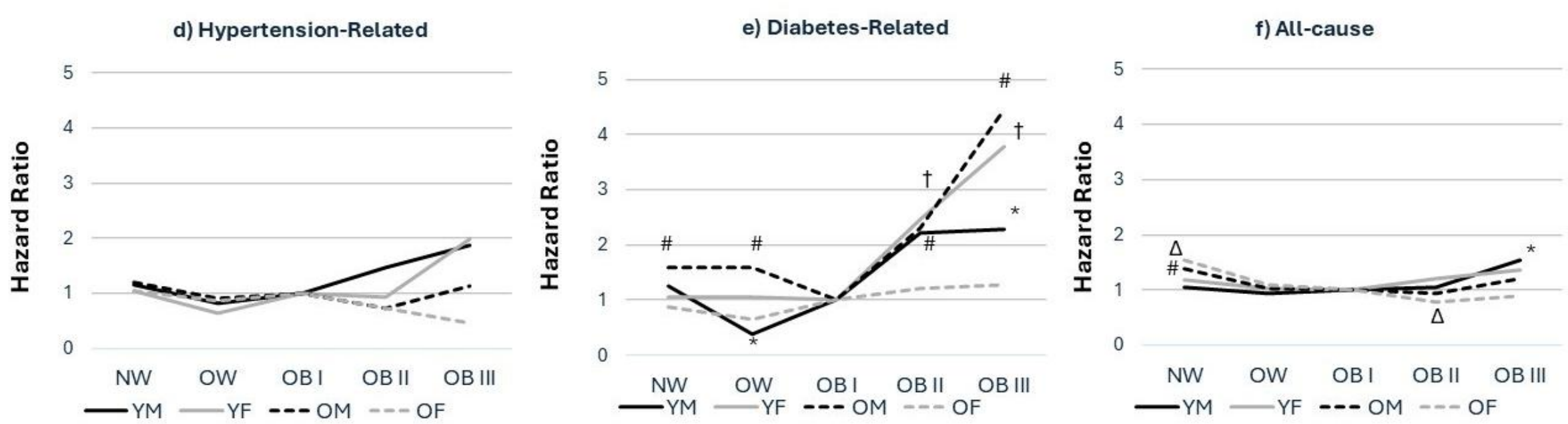

d) Hypertension-Related

	NW	OW	OB I	OB II	OB III
YM	1.1 (0.5 - 2.9)	0.8 (0.3 - 1.9)	1 (. - .)	1.5 (0.5 - 4.8)	1.9 (0.7 - 4.9)
YF	1.0 (0.4 - 2.8)	0.6 (0.3 - 1.6)	1 (. - .)	0.9 (0.4 - 2.5)	2.0 (1.0 - 4.1)
OM	1.2 (0.8 - 1.8)	0.9 (0.6 - 1.3)	1 (.-.)	0.7 (0.4 - 1.3)	1.1 (0.5 - 2.5)
OF	1.0 (0.7 - 1.5)	0.9 (0.6 - 1.2)	1 (.-.)	0.7 (0.5 - 1.2)	0.5 (0.2 - 1.0)

Hazard Ratio (95% CI)

e) Diabetes-Related

	NW	OW	OB I	OB II	OB III
YM	1.3 (0.6 - 2.7)	**0.4 *** (0.2 - 0.8)	1 (. - .)	2.2 (0.9 - 5.6)	**2.3 *** (1.1 - 4.9)
YF	1.0 (0.5 - 2.3)	1.1 (0.5 - 2.3)	1 (. - .)	**2.5 †** (1.1 – 5.5)	**3.8†** (1.8 - 8.1)
OM	**1.6#** (1.02 - 2.5)	**1.6#** (1.1 - 2.4)	1 (. - .)	**2.3#** (1.2 - 4.6)	**4.4#** (2.0 - 9.9)
OF	0.9 (0.5 - 1.5)	0.7 (0.4 - 1.1)	1 (. - .)	1.2 (0.7 - 2.1)	1.3 (0.7 - 2.5)

Hazard Ratio (95% CI)

f) All-cause

	NW	OW	OB I	OB II	OB III
YM	1.1 (0.8 - 1.4)	0.9 (0.7 - 1.2)	1 (. - .)	1.1 (0.8 - 1.5)	**1.5 *** (1.1 - 2.2)
YF	1.2 (0.8 - 1.7)	1.0 (0.7 - 1.5)	1 (. - .)	1.2 (0.8 – 1.8)	1.4 (1.0 – 1.9)
OM	**1.4#** (1.2 - 1.7)	1.0 (0.9 - 1.2)	1 (. - .)	0.9 (0.7 - 1.2)	1.2 (0.7 - 2.0)
OF	**1.5 Δ** (1.3 - 1.8)	1.1 (0.9 - 1.3)	1 (. - .)	**0.8 Δ** (0.6 - 0.97)	0.9 (0.7 - 1.2)

Hazard Ratio (95% CI)

Values represent adjusted hazard ratios adjusted for ethnicity, education, smoking, physical activity and medication use. NW= Normal weight. OW= Overweight. OB I= Obesity class I. OB II= Obesity class II. OB III= Obesity class III. YM= Younger males. YF= Younger Females. OM= Older males. OF= Older females. *= significant difference from younger males (18-64 years) with obesity class I. †= significant difference from younger females with obesity class I. #= significant difference from older males ($\geq$65 years) with obesity class I. Δ= significant difference from older females with obesity class I.
